# A digital audit of emergency upper gastrointestinal fluoroscopy workflow in children with bilious vomiting

**DOI:** 10.4102/sajr.v26i1.2300

**Published:** 2022-03-30

**Authors:** Bradley C. Messiahs, Richard D. Pitcher

**Affiliations:** 1 Division of Radiodiagnosis, Department of Medical Imaging and Clinical Oncology, Faculty of Medicine and Health Sciences, Stellenbosch University, Cape Town, South Africa

**Keywords:** bilious, vomiting, malrotation, midgut volvulus, upper gastrointestinal series, paediatric

## Abstract

**Background:**

Bilious vomiting in children requires an urgent evaluation with upper gastrointestinal (UGI) fluoroscopy as it may herald life-threatening midgut malrotation with volvulus (MMWV). There are no published data available on the duration of time-critical UGI workflow steps.

**Objectives:**

A digital audit of workflow in emergency UGI contrast studies performed on children with bile-stained vomiting at a large South African teaching hospital.

**Method:**

A retrospective study was conducted from 01 May 2012 – 31 May 2019. A customised search of the institutional radiology information system (RIS) defined all children with bilious vomiting who underwent emergency UGI fluoroscopy. Extracted RIS timestamps were used to calculate the median duration of the ‘approval’, ‘waiting’, ‘study’ and ‘reporting’ times. One-way analysis of variance and Chi-squared tests assessed the association between key parameters and the duration of workflow steps, with 5% significance (*p* < 0.05).

**Results:**

Thirty-seven patients (*n* = 37) with median age 0.8 months were included, of whom 20 (54%) had an abnormal C-loop. The median ‘total time’ from physician request to report distribution was 107 min (interquartile range [IQR]: 67−173). The median ‘approval’ (6 min; IQR: 1–15) and ‘reporting’ (38 min; IQR: 17–91) times were the shortest and longest workflow steps, respectively. Abnormal C-loops (*p* = 0.04) and consultant referrals (*p* = 0.03) were associated with shorter ‘approval’ times. The neonatal ‘waiting’ time was significantly longer than that for older patients (*p* = 0.02).

**Conclusion:**

The modern RIS is an excellent tool for time-critical workflow analyses, which can inform interventions for improved service delivery.

## Introduction

Vomiting in childhood should be categorised clinically as either bile-stained (bilious) or non-bile-stained (non-bilious). The differentiation may be challenging, but is crucial. Non-bilious vomiting is commonly innocuous and occurs as a result of gastro-esophageal reflux while bile-stained vomiting is typically caused by partial or complete bowel obstruction distal to the ampulla of Vater.^1^

Bilious vomiting in the paediatric age-group requires urgent evaluation. It may herald life-threatening midgut malrotation with volvulus (MMWV) of the intestine about the superior mesenteric artery (SMA) and associated bowel ischaemia or necrosis.

Intestinal malrotation is present in approximately one in 500 live births.^2^ Normal intestinal rotation occurs in the 10th week of gestation as the bowel migrates back into the abdominal cavity following a brief period at the base of the umbilical cord. As the intestine returns to the abdominal cavity, it makes two rotations and becomes fixed into its normal position, with the colon draped lateral and superior to the centrally located small intestine.^3^

Malrotation results from incomplete intestinal rotation, and failure of fixation. As a result, the large intestine lies on the left side of the abdominal cavity and the small intestine on the right. The caecum and appendix, normally fixed posteriorly in the right lower abdomen, are free and located centrally in the mid-upper abdomen. The duodenum, normally attached dorsally across the midline in the upper abdomen, is also not fixed and typically lies in the right upper quadrant of the abdomen. The root of the small bowel mesentery is thus narrow, and prone to twist around the SMA and superior mesenteric vein (SMV).^4^ This twisting or ‘volvulus’ of small bowel on its own blood supply may result in ischaemia and ultimately necrosis. Seventy-five per cent of symptomatic cases of malrotation occur in neonates, and up to 90% of such cases occur within the 1st year of life.^2,5,6^

Whilst MMWV most commonly presents in the neonatal period, it can present at any time during childhood, although the frequency decreases with increasing age.^7^ The classical clinical manifestation of malrotation with volvulus is bilious vomiting.^5,8,9^ Among children with bile-stained vomiting in the first 72 h of life, approximately one-fifth required surgical intervention.^7^

**FIGURE 1:**
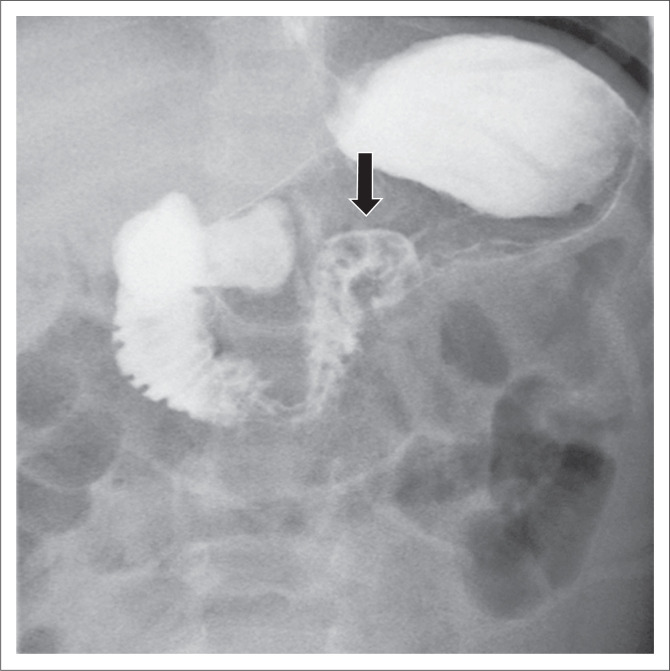
Normal upper gastrointestinal series in an infant with vomiting. Fluoroscopic frontal view shows the duodenojejunal junction (arrow) to the left of a vertebral body pedicle and at the level of the duodenal bulb.

Mortality in neonates with MMWV was as high as 30% as recently as the 1960s^10,11^ but has subsequently decreased to approximately 3% − 5%.^9,12,13^ Because of the life-threatening potential of MMWV, this must be excluded as a matter of urgency in any child presenting with bile-stained vomiting. Upper gastrointestinal (UGI) fluoroscopy is the examination of choice. It is performed to assess the position of the duodeno-jejunal (DJ) flexure.^14^ On the frontal projection, the normal DJ flexure lies lateral to the left pedicle of the vertebral body adjacent to the duodenal bulb (Figure 1).^15^

The demonstration of a normally positioned DJ flexure implies normal fixation of the root of the mesentery and does not indicate intestinal malrotation. Conversely, failure to demonstrate the DJ flexure in a normal position warrants immediate surgical exploration of the abdomen.^16^ The performance of emergency UGI fluoroscopy for bile-stained vomiting in childhood is thus considered both mandatory and time-critical. It should be performed as the first investigation and as soon as possible, since delayed diagnosis can be life-threatening, while early diagnosis has an excellent prognosis.^14^ A water soluble, low osmolality contrast medium is preferred.^17,18^

Despite the urgency of UGI contrast studies in this clinical setting, to the best of our knowledge, there are no published data on the duration of workflow steps. The modern digital radiology department, with an integrated picture-archiving and communication system/radiology information system (PACS/RIS) provides the ideal platform for conducting such audits. The integrative functions of the modern PACS/RIS include the capacity to provide timestamps for each step in the digital imaging workflow.^19^ Additionally, there has been no detailed description of the fluoroscopic findings in children with bilious vomiting in our setting.

The primary aim of this study was a digital audit of time-critical workflow steps in emergency UGI contrast studies performed on children with bile-stained vomiting at a large South African (SA) teaching hospital. The secondary aim was a description of the fluoroscopic findings in a cohort of SA children with bile-stained vomiting.

## Methods

This retrospective, descriptive study was conducted at Tygerberg Hospital (TBH), Cape Town, South Africa. Tygerberg Hospital is a public sector facility with a fully digital, paperless radiology department, including an integrated PACS/RIS. Physician requests for imaging are entered electronically and require the approval and assignment of acuity by the radiologist on duty. All clinical data recorded in the imaging request are archived and amenable to interrogation.

A customised search of the TBH/RIS was conducted, utilising embedded data mining software. The study covered the period from 01 May 2012 to 31 May 2019. All children with the term ‘bile-stained vomiting’ or ‘bilious vomiting’ in the clinical history on the electronic imaging request, and who subsequently underwent emergency UGI fluoroscopy, were included. Children without a history of bilious vomiting, those who did not undergo an emergency UGI contrast study and all patients 18 years or older, were excluded. For each subject, the digital RIS records pertaining to the emergency UGI study were interrogated and the following timestamps retrieved: (1) physician request, (2) radiologist approval, (3) start of study, (4) study conclusion and (5) report completion.

For all included studies, patient demographics (age, gender), referral details (rank of referring doctor, referring specialty), service parameters (request time and day, seniority of radiology registrar), radiological findings (normal or abnormal C-loop) and key PACS/RIS workflow timestamps were captured on a customised Excel spreadsheet. Statistica (www.statsoft.com), the software suite for data analytics, was used for performing data analysis.

The normal working hours were defined as 08:00–16:00 Monday to Friday. Investigations performed outside these times were deemed after-hour procedures. Junior registrars were defined as registrars with less than two years of accredited training time.

- The following workflow steps were analysed: (1) approval time: Clinician request to radiologist approval, (2) waiting time: Radiologist approval to start of study, (3) study time: Study start to end, (4) reporting time: Study completion to report distribution and (5) total time: Clinician request to report distribution.

Descriptive statistics were generated by using the extracted RIS timestamps to calculate the median time and interquartile range (IQR) for each workflow step. The one-way analysis of variance (ANOVA) *F*-test and the chi-square tests were used to assess any associations between demographic factors, referral details, service parameters, radiological findings and prolongation of workflow steps, with a 5% significance level (*p* < 0.05).

### Ethical considerations

The study was approved by the Health Research Ethics Committee of the Faculty of Medicine and Health Sciences, Stellenbosch University (Ref: S20/03/084). Confidentiality was maintained throughout the study.

## Results

A total of 37 patients with a median age of 0.8 months (IQR: 0.1–9.0 months) were included, of whom almost two-thirds (24/37; 65%) were neonates. Approximately 90% of referrals (33/37; 89%) were from the departments of paediatrics (18/37; 49%) or paediatric surgery (15/37; 41%) and more than 80% (30/37, 81%) were from consultants (16/37; 43%) or registrars (14/37; 37%). The studies were performed by radiology registrars with a median of 1.9 (IQR: 1.0–2.8) years of accredited training time; almost two-thirds of investigations (23/37, 62%) were conducted outside normal working hours.

Just over a half of the cohort (20/37; 54%) had an abnormal C-loop, with subsequent emergency laparotomy. Intraoperative findings included malrotation with volvulus (7/20; 35%), malrotation without volvulus (3/20; 15%), high grade proximal jejunal obstruction (4/20; 20%), duodenal atresia (2/20; 10%) and duodenal webs (2/20; 10%).

The median times, with IQRs, for the UGI workflow steps for the whole group, and by imaging, patient, operator and time parameters are documented in Tables 1–5. The median ‘total time’ (Table 1), from physician request to report distribution was 107 min (IQR: 67−173). Although the ‘approval time’ was the shortest workflow step (6 min; IQR: 1−15), it was also the most variable, with a 1:15 ratio between the first and third quartiles. The ‘reporting time’ was the longest step (38 min; IQR: 17−91) and showed moderate variability, with a 1:5 ratio between the first and third quartiles. The median ‘waiting time’ of 28 min (IQR: 20−52) was the most consistent, with a 1:2.6 ratio between the first and third quartiles.

**TABLE 1:** Whole group.

Workflow steps	Approval time	Waiting time	Study time	Reporting time	Total time
Whole group	6	28	9	38	107
IQR	1–15	20–52	4–13	17–91	67–173

Note: Values are given in minutes.

IQR, interquartile range.

**TABLE 2:** Imaging parameters.

Workflow steps	Approval time	IQR	*p*	Waiting time	IQR	*p*	Study time	IQR	*p*	Reporting time	IQR	*p*	Total time	IQR	*p*
Normal C-loop	11	3–23	0.04	27	20–38	0.86	8	5–11	0.09	27	17–106	0.35	95	56–169	0.48
Abnormal C-loop	2	1–12	-	37	22–66	-	9	4–24	-	50	17–96	-	123	70–196	-

Note: Values are given in minutes.

IQR, interquartile range.

**TABLE 3:** Patient parameters.

Workflow steps	Approval time	IQR	*p*	Waiting time	IQR	*p*	Study time	IQR	*p*	Reporting time	IQR	*p*	Total time	IQR	*p*
Neonate (≤ 1 month)	6.5	1–15	0.94	37	25–62	0.02	7	3–12	0.13	44	15–114	0.80	117	70–196	0.38
Non-neonate (> 1 month)	6	2–17	-	24	18–29	-	11	8–23	-	27	18–91	-	106	62–156	-

Note: Values are given in minutes.

IQR, interquartile range.

**TABLE 4:** Radiologist parameters.

Workflow steps	Approval time	IQR	*p*	Waiting time	IQR	*p*	Study time	IQR	*p*	Reporting time	IQR	*p*	Total time	IQR	*p*
< 2 years’ experience	8	2–15	0.30	29	24–52	0.56	9	3–12	0.89	46	17–137	0.74	137	69–210	0.49
> 2 years’ experience	6	1–15	-	27	16–60	-	8	4–14	-	38	14–62	-	106	60–140	-

Note: Values are given in minutes.

IQR, interquartile range.

**TABLE 5:** Time parameters.

Workflow steps	Approval time	IQR	*p*	Waiting time	IQR	*p*	Study time	IQR	*p*	Reporting time	IQR	*p*	Total time	IQR	*p*
Normal hours	5	1–12	0.78	22	16–47	0.08	11	6–23	0.35	59	18–137	0.48	132	67–173	0.45
After hours	6	1–15	-	31	24–60	-	8	3–12	-	28	17–74	-	93	63–181	-

Note: Values are given in minutes.

IQR, interquartile range.

The ‘approval time’ (Table 2) of 2 min (IQR: 1−12) for studies ultimately demonstrating an abnormal C-loop was significantly shorter than that for studies subsequently reported as normal (11 min; IQR: 3−23; *p* = 0.04). The ‘waiting time’ (Table 3) for neonates (37 min; IQR: 25−62) was significantly longer than that for older patients (24 min; IQR: 18−29; *p* = 0.02).

The median approval time for consultant referrals (2 min; IQR: 1−6) was significantly shorter than that for registrars (12 min; IQR: 3−20), medical officers (14 min; IQR: 6−27) and interns (25 min; IQR: 7−43; *p* = 0.03), but there was no association between the waiting time (*p* = 0.30), reporting time (*p* = 0.73) or total time (*p* = 0.42) and the level of the referring clinician.

Although not statistically significant, the performance of junior registrars was slower at each step of the workflow (Table 4).

## Discussion

To the best of our knowledge, this is the first audit of time-critical workflow steps in the performance of emergency UGI studies for children with bile-stained vomiting in any setting. This study highlights the pivotal role of the modern RIS in enabling a detailed analysis of the digital imaging workflow. By providing baseline temporal data on the completion of emergency paediatric UGI studies for bile-stained vomiting, it makes an important contribution to radiological quality assurance in this setting. There are several key findings.

Firstly, the median ‘total time’ from physician request to report completion was just under 2 h (107 min; IQR = 67–173). This must be viewed in the context of the estimated 8 h − 16 h required for bowel ischaemia to progress to complete transmural infarction.^20^ In the face of mesenteric ischaemia one has just 8 h − 16 h for correct diagnosis and management, to prevent bowel necrosis. Beyond this period, bowel resection is the only treatment option, with prognosis dependent on the extent of resection.^20^ Considering the late presentation of many patients in our environment, the duration of diagnostic work-up must be kept to the absolute minimum. It is thus beneficial to adopt the same approach as for radiation exposure, and to commit to achieving work-up times ‘as low as reasonably achievable’, the so-called ‘ALARA’ principle,^21^ but in a different context. Similarly, one should adopt the mindset invoked for the acute stroke patient, where ‘time is brain’.^22^ In this instance ‘time is gut’.

Secondly, the IQR for the ‘total time’ was between 67 min and 173 min. This suggests that there is considerable room for improvement, since imaging work-up for the first quartile of our cohort was largely completed in less than 1 h (67 min), while that for the fourth quartile mostly exceeded 3 h (173 min). The purpose of workflow studies such as this is to define these key parameters. We have shown that in a sizable minority of cases it is possible to complete imaging work-up within an hour. Such knowledge benchmarks diagnostic timeframes going forward. To define a strategy for improved performance, each workflow step must be reviewed.

Since the IQR reflects the data spread, the ratio between IQR values (1st vs. 3rd) indicates the consistency of performance for each step. The 1:15 IQR ratio for ‘approval time’ indicates substantial variability. This should prompt critical analysis with a view to achieving greater consistency. Conversely, the 1:2.6 IQR ratio for ‘waiting time’ indicates relative consistency, although this does not necessarily imply optimal performance, since it may simply indicate entrenched inefficiency. It is in this context that workflow steps are analysed.

The wide variability in the ‘approval time’ suggests deviation from the standard TBH workflow for emergency imaging investigations. These stipulate that referring clinicians should make direct telephonic contact with the radiologist on duty for digital approval of emergency procedures immediately after entering the digital request. The finding that the median ‘approval time’ was 6 min, and the IQR 1 min − 15 min, suggests that standard workflow was followed in just one-quarter of cases, as shown by an ‘approval time’ of less than 1 min in such instances. This finding also suggests that simply entrenching existing TBH workflow guidelines would substantially enhance performance in this step. We found the ‘approval time’ for consultant referrals significantly shorter than those for other staff. This indicates closer adherence to institutional workflow guidelines in such instances and that consultant telephonic requests for emergency investigations elicit a more rapid radiological response. We also found that ‘approval time’ for studies with an abnormal C-loop was significantly shorter than for studies subsequently considered normal. This suggests that the clinical findings in cases with an abnormal C-loop were more compelling, with features of greater acuity, prompting swifter responses by role players. The referring clinician would, in such instances, be more inclined to discuss the need for emergency imaging with the radiologist on duty.

An analysis of ‘waiting time’ is similarly informative. We showed that the median time from study approval to commencement was approximately half-an-hour (28 min). However, for the first quartile, this was within 20 min, and for the fourth quartile, longer than 52 min. Furthermore, the waiting time for neonates was significantly longer than for older subjects. The resuscitation of unstable patients may account for these findings. The performance of this step could potentially be improved by requesting the referring physicians to personally accompany patients to the fluoroscopy suite, without recourse to porters and trolleys.

Although not statistically significant, we found that the study time for the abnormal C-loop tended to be longer, and showed greater variability, than that for the normal C-loop.

This is understandable since the abnormal C-loop may manifest as duodenal obstruction with delayed transit of contrast and prolongation of the examination. In a recent report on a small patient cohort, the standard UGI contrast study was modified to ‘selective duodenography’, whereby a nasogastric tube was purposefully advanced to lie in the proximal duodenum. Approximately 10 mL of water-soluble contrast medium was introduced via the nasogastric tube, with careful control of the timing, pressure and volume, thereby allowing optimal duodenal opacification.^23^ Although the initial findings suggest that this technique yields good results, duodenal intubation is not always straightforward and may be time-consuming. Further comparative work is required in a larger, controlled study to define any advantages with respect to the overall workflow duration.

More than a half of our cohort with bilious vomiting (20/37; 54%) were shown to have a surgical cause. The reported proportion of neonates with bilious vomiting who are subsequently diagnosed with surgical pathology varies widely (13% − 50%) in the literature.^24,25,26,27^ We thus found a relatively high proportion of patients with surgical pathology compared to the international literature. However, the wide variation in the proportion of patients with surgical pathology documented in published work likely reflects variable inclusion criteria for the individual studies.

A major strength of this study is its foundation on an exceptionally robust database, namely the institutional RIS, which yields comprehensive and accurate details, thereby ensuring the integrity of the findings. It is also the first study of its kind to utilise the RIS for workflow analysis in this clinical setting. The study limitations were its retrospective nature and small sample size.

We hope that this work prompts similar workflow analyses in other institutions and provides a benchmark for subsequent improvement within our institution. Specifically, referring clinicians should be encouraged to discuss these cases telephonically with the radiologist on duty and to accompany patients to the radiology department to minimise delays.

## Conclusion

The modern RIS is an excellent tool for time-critical workflow analyses, which can, in turn, inform interventions for improved service delivery.

## References

[1] Alazraki AL, Rigsby CK, Iyer RS, et al. ACR appropriateness criteria: Vomiting in infants. J Am Coll Radiol. 2020;17(11S):S505–S515. 10.1016/j.jacr.2020.09.002 33153561

[2] Torres AM, Ziegler MM. Malrotation of the intestine. World J Surg. 1993;17(3):326–331. 10.1007/BF01658699 8337878

[3] Intestinal malrotation and volvulus [homepage on the Internet]. c2019 [updated 2019 July; cited 2021 Sep 5]. Available from: https://www.cincinnatichildrens.org/health/i/intestinal-malrotation

[4] Applegate KE, Anderson JM, Eugene C. Intestinal malrotation in children: A problem-solving approach to the upper gastrointestinal series. Radiographics. 2006;26(5):1485–1500. 10.1148/rg.265055167 16973777

[5] Strouse P. Disorders of intestinal rotation and fixation (‘malrotation’). Pedriatic Radiol. 2004;34(11):837–851. 10.1007/s00247-004-1279-4 15378215

[6] Spigland N, Brandt ML, Yazbeck S. Malrotation presenting beyond the neonatal period. J Pediatr Surg. 1990;25:1139–1142. 10.1016/0022-3468(90)90749-y 2273427

[7] Lilien LD, Srinivasan G, Pyati SP, et al. Green vomiting in the first 72 hours in normal infants. Am J Dis Child. 1986;140(7):662–664. 10.1001/archpedi.1986.02140210060026 3717104

[8] Stringer DA, Babyn PS. Pediatric gastrointestinal imaging and intervention [homepage on the Internet]. Volume 1., 2nd ed. Hamilton: Decker, 2000 [cited 2021 Sep 5]; p. 816. Available from: https://books.google.com/books?id=n6MSJLPKJTsC&pgis=1

[9] Bonadio WA, Clarkson T, Naus J. The clinical features of children with malrotation of the intestine. Pediatr Emerg Care. 1991;7:348–349. 10.1097/00006565-199112000-00007 1788123

[10] Rescorla FJ, Shedd FJ, Grosfeld JL, et al. Anomalies of intestinal rotation in childhood: Analysis of 447 cases. Surgery. 1990;108(4):710–716. 2218883

[11] Berdon WE, Baker DH, Bull S. Midgut malrotation and volvulus. Radiology. 1970;96:375–383. https://doi.org/1011.48/96.2.375 5431424 10.1148/96.2.375

[12] Andrassy RJ, Mahour GH. Malrotation of the midgut in infants and children: A 25-year review. Arch Surg. 1981;116(2):158–160. https://doi.org/101001/archsurg.1981.01380140020004 7469743 10.1001/archsurg.1981.01380140020004

[13] Ford EG, Senac MO Jr, Srikanth MS, et al. Malrotation of the intestine in children. Ann Surg. 1992;215:172–178. 10.1097/00000658-199202000-00013 1546904 PMC1242406

[14] Drewett M, Johal N, Keys C, et al. The burden of excluding malrotation in term neonates with bile stained vomiting. Pediatr Surg Int. 2016;32(5):483–486. 10.1007/s00383-016-3877-2 26895031

[15] Long FR, Kramer SS, Markowitz RI, et al. Intestinal malrotation in children: Tutorial on radiographic diagnosis in difficult cases. Radiology. 198(3):775–780. 10.1148/radiology.198.3.8628870 8628870

[16] Malhotra A, Lakkundi A, Carse E. Bilious vomiting in the newborn: 6 years data from a level III centre. J Paediatr Child Health. 2010;46(5):259–261. 10.1111/j.1440-1754.2009.01681.x 20337876

[17] Omnipaque [homepage on the Internet]. c2017 [updated 2017 March; cited 2021 Sep 5]; p. 1–39. Available from: https://www.accessdata.fda.gov/drugsatfda_docs/label/2017/018956s099lbl.pdf

[18] Radiological Society of South Africa/South African Society of Paediatric Imaging (RSSA/SASPI). Paediatric imaging guidelines, Radiological Society of South Africa, Tokai, South Africa. 2013 Jan 10, version 1.1; p. 11–13.

[19] Crabbe JP, Frank CL, Nye WW. Improving report turnaround time: An integrated method using data from a radiology information system. Am J Roentgenol. 1994;163(6):1503–1507. 10.2214/ajr.163.6.7992756 7992756

[20] Haglund U. Mesenteric ischemia. In: Holzheimer RG, Mannick JA, editors. Surgical treatment: Evidence-based and problem-oriented [homepage on the Internet]. Munich: Zuckschwerdt; 2001 [cited 2021 Sep 5]. Available from: https://www.ncbi.nlm.nih.gov/books/NBK6883/21028753

[21] Strauss KJ, Kaste SC. ALARA in pediatric interventional and fluoroscopic imaging: Striving to keep radiation doses as low as possible during fluoroscopy of pediatric patients – A white paper executive summary. J Am Coll Radiol. 2006;3(9):686–688. 10.1016/j.jacr.2006.04.008 17412149

[22] Saver JL. Time is brain – Quantified. Stroke. 2006;37(1):263–266. 10.1161/01.STR.0000196957.55928.ab 16339467

[23] Andronikou S, Arthur S, Simpson E, et al. Selective duodenography for controlled first-pass bolus distention of the duodenum in neonates and young children with bile-stained vomiting. Clin Radiol. 2018;73(5):506.e1–506.e8. 10.1016/j.crad.2017.12.020 29397912

[24] Mohinuddin S, Sakhuja P, Bermundo B, et al. Outcomes of full-term infants with bilious vomiting: Observational study of a retrieved cohort. Arch Dis Child. 2015;100(1):14–17. 10.1136/archdischild-2013-305724 25204734

[25] Godbole P, Stringer MD. Bilious vomiting in the newborn: How often is it pathologic? J Pediatr Surg. 2002;37(6):909–911. 10.1053/jpsu.2002.32909 12037761

[26] Ojha S, Sand L, Ratnavel N, et al. Newborn infants with bilious vomiting: A national audit of neonatal transport services. Arch Dis Child Fetal Neonatal Ed. 2017;102(6):F515–F518. 10.1136/archdischild 28483818

[27] Lee RA, Dassios T, Bhat R, et al. A. Bilious vomiting in the newborn: A three-year experience in a tertiary medical and surgical centre. Case Rep Pediatr. 2020;2020:8824556. 10.1155/2020/8824556 33110665 PMC7578730

